# Comparison of the RF-CL and CACS-CL models to estimate the pretest probability of obstructive coronary artery disease and predict prognosis in patients with stable chest pain and diabetes mellitus

**DOI:** 10.3389/fcvm.2024.1368743

**Published:** 2024-03-22

**Authors:** Tao Chen, Dujing Shao, Jia Zhao, Mingwen Xiu, Yaoshuang Li, Miao He, Yahang Tan, Yanchun An, Xiangchen Zhang, Jia Zhao, Jia Zhou

**Affiliations:** ^1^ Department of Emergency, Hebei Petrochina Central Hospital, Langfang, Hebei, China; ^2^ Department of Cardiology, Tianjin Chest Hospital, Tianjin, China; ^3^ Department of Cardiology, Beijing Chaoyang Hospital, Capital Medical University, Beijing, China; ^4^ Heart Center and Beijing Key Laboratory of Hypertension, Beijing Chaoyang Hospital, Capital Medical University, Beijing, China; ^5^ Department of Radiology, Hebei Petrochina Central Hospital, Langfang, Hebei, China

**Keywords:** diabetes mellitus, stable chest pain, coronary computed tomography angiography, risk assessment, coronary artery calcium score, pretest probability

## Abstract

**Background:**

The most appropriate tool for estimating the pretest probability (PTP) of obstructive coronary artery disease (CAD) in patients with diabetes mellitus (DM) and stable chest pain (SCP) remains unknown. Therefore, we aimed to validate and compare two recent models, namely, the risk factor-weighted clinical likelihood (RF-CL) model and coronary artery calcium score (CACS)-weighted clinical likelihood (CACS-CL) model, in these patient populations.

**Methods:**

A total of 1,245 symptomatic patients with DM, who underwent CACS and coronary computed tomographic angiography (CCTA) scan, were identified and followed up. PTP of obstructive CAD for each patient was estimated using the RF-CL model and CACS-CL model, respectively. Area under the receiver operating characteristic curve (AUC), net reclassification improvement (NRI), and integrated discrimination improvement (IDI) were used to assess the performance of models. The associations of major adverse cardiovascular events (MACE) with risk groups were evaluated using Cox proportional hazards regression.

**Results:**

Compared with the RF-CL model, the CACS-CL model revealed a larger AUC (0.856 vs. 0.782, *p* = 0.0016), positive IDI (12%, *p* < 0.0001) and NRI (34%, *p* < 0.0001), stronger association to MACE (hazard ratio: 0.26 vs. 0.38) and less discrepancy between observed and predicted probabilities, resulting in a more effective risk assessment to optimize downstream clinical management.

**Conclusion:**

Among patients with DM and SCP, the incorporation of CACS into the CACS-CL model resulted in a more accurate estimation for PTP and prediction of MACE. Utilizing the CACS-CL model, instead of the RF-CL model, might have greater potential to avoid unnecessary and omissive cardiovascular imaging testing with minimal cost.

## Background

The number of patients with diabetes mellitus (DM) is expected to increase to 700 million by 2045 worldwide (1). The growing worldwide burden of impaired glucose metabolism coupled, with the high rates of coronary artery disease (CAD) morbidity and mortality among affected individuals, represents an important priority for patients and health systems (2, 3). Although DM was previously considered a CAD equivalent, the average cardiovascular risk in symptomatic individuals with DM varies significantly depending on other risk factors (4, 5). This heterogeneity in patients with stable chest pain (SCP) and DM poses a unique challenge for clinicians tasked with effective risk assessment and selection of appropriate cardiovascular imaging testing (CIT) from an ever-growing list of options, such as coronary computed tomographic angiography (CCTA).

In contrast to the “screen all” strategy, which was not supported by contemporary evidence (6, 7), the 2023 European Society of Cardiology (ESC) guidelines for the management of cardiovascular disease in patients with DM (2) proposed an estimation for pretest probability (PTP) of CAD based on the 2019 ESC guidelines on chronic coronary syndromes (CCS) (8). Our previous study based on data from the CCTA Improves Clinical Management of Stable Chest Pain (CICM-SCP) registry has demonstrated that the application of the risk factor-weighted clinical likelihood (RF-CL) model was associated with improved efficiency in identifying individuals who may derive maximum benefit from further CIT in patients with DM and SCP (9).

In addition to the RF-CL model, another coronary artery calcium score (CACS)-based model was also developed for the estimation of PTP, and several external validations in general SCP patients overwhelmingly supported the CACS-weighted clinical likelihood (CACS-CL) model (10–13). Notably, both the RF-CL and CACS-CL models were recommended by the newest guideline for the evaluation and diagnosis of SCP (14). However, to date, no comparative analysis has been conducted to systematically evaluate the RF-CL and CACS-CL models in DM patients with SCP, for whom the appropriate decision-making of CIT is important but difficult (1).

Thus, we aim to validate and compare the two proposed models in a CCTA-based cohort comprised of DM patients presenting with SCP and investigate whether the addition of CACS would improve the effectiveness of risk assessment to optimize downstream clinical management.

## Methods

### Study cohort

**Figure 1:**
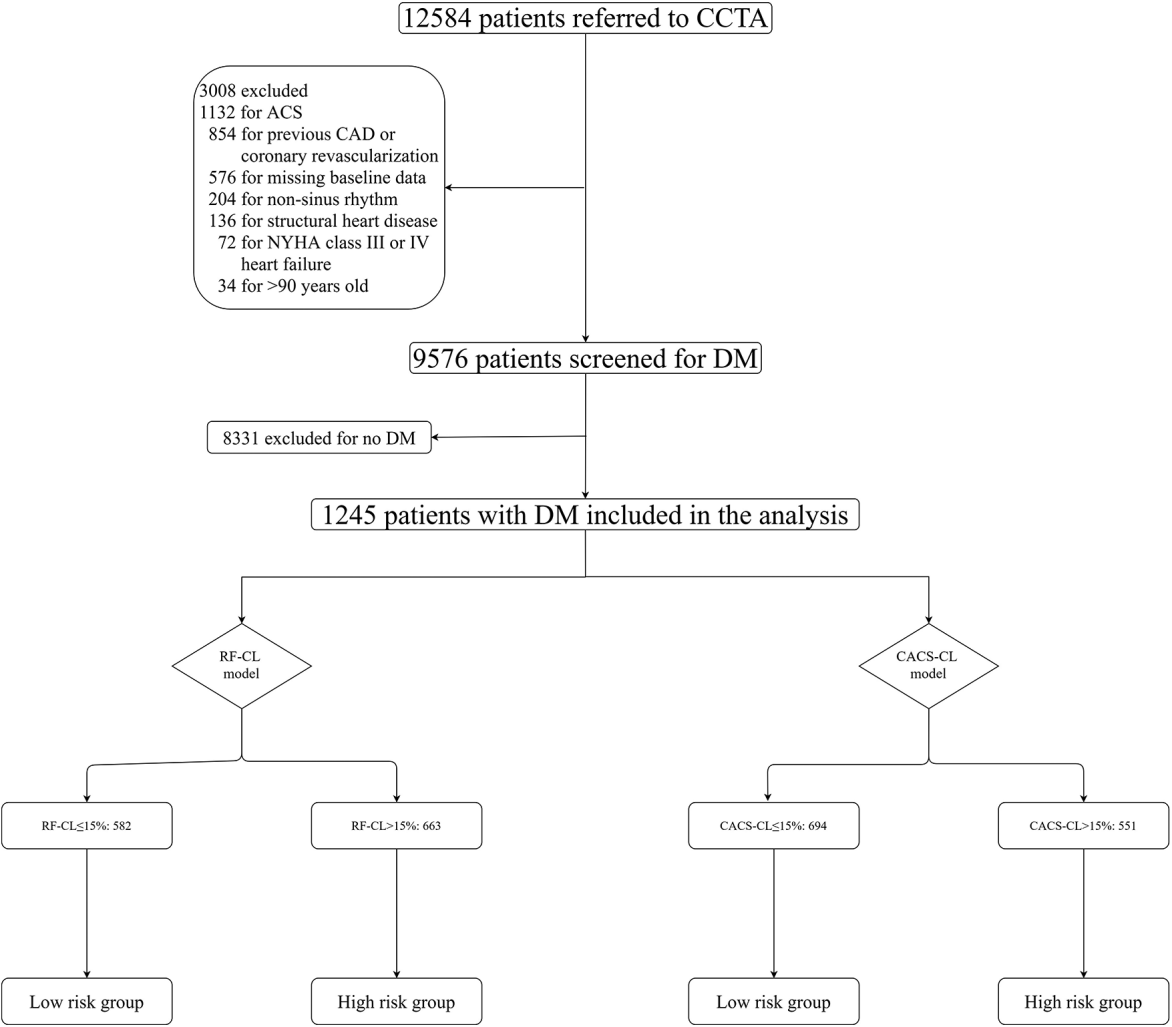
Flowchart. CCTA, coronary computed tomographic angiography; RF-CL, risk factor-weighted clinical likelihood; CACS-CL, coronary artery calcium score-weighted clinical likelihood; CAD, coronary artery disease; ACS, acute coronary syndrome.

Briefly, the CICM-SCP registry is a prospective and ongoing cohort of patients who were referred to CCTA as first-line imaging testing for the assessment of SCP suspected of CCS (ClinicalTrials.gov Identifier: NCT04691037). Details about the registry have been previously described (11, 12, 15–18) and are further provided in the Supplementary Material. As illustrated in Figure 1, during a period of 36 months between August 2016 and August 2019, 12,584 patients with scans of both CACS and CCTA were initially enrolled, and 9,576 patients were screened for DM. The patients were considered as suffering from DM if one of the following was met: treatment with insulin or hypoglycemic medications, fasting blood glucose ≥7.0 mmol/L or a 2 h plasma glucose level on their oral glucose tolerance test ≥11.1 mmol/L, or a glycated hemoglobin value ≥6.5%. Finally, 1,245 patients with DM and SCP were included in the present analysis. The present study was conducted in accordance with the Declaration of Helsinki and approved by the ethics committees of local institutions.

### Collection and definitions of baseline characteristics

Except for DM, baseline clinical data including age, sex, hypertension, hyperlipidemia, smoking, family history of CAD, and type of SCP were prospectively collected and defined as described previously in the CICM-SCP registry (11, 12, 15–18). The procedure and interpretation details of CACS and CCTA have been previously described (11, 15, 17). These details are also provided in the Supplementary Material. Obstructive CAD was defined as present if a patient had at least one lesion with ≥50% diameter stenosis or any unassessable segments because of severe calcification on CCTA.

### PTP models

The RF-CL model (age, sex, type of SCP, DM, hypertension, hyperlipidemia, family history, and smoking) and CACS-CL model (age, sex, type of SCP, DM, hypertension, hyperlipidemia, family history, smoking, and CACS) were used to estimate the PTP of obstructive CAD as previously reported, and the R packages are available at https://github.com/CardioLab/cadptp/tree/master/R (10). According to the current guidelines (8, 14) and our previous works (11, 19), CIT should be deferred for a low-risk patient, and the impact of PTP on outcome was tested by classifying patients into different risk groups. The details of the risk groups are illustrated in Figure 1, and according to the RF-CL and CACS-CL models, patients with PTP ≤ 15% were divided into the low-risk group, and patients with PTP > 15% were divided into the high-risk group.

### Follow-up and clinical outcomes

The details about the follow-up have been described previously in the CICM-SCP registry (11, 12, 15–18) and are also provided in the Supplementary Material. After CCTA, all patients were followed until March 2023. The primary endpoint was a major adverse cardiovascular event (MACE), which was defined as a composite of cardiac death and non-fatal myocardial infarction (MI). Cardiac death was defined as any death caused by cardiac disease or for which no other cause could be found. MI was defined as described in the Fourth Universal Definition of Myocardial Infarction (20). The secondary endpoint included ICA utilization and referral to revascularization. For a patient undergoing repeat invasive procedures, we mainly focused on the first utilization of invasive procedures (21–23).

### Statistical analyses

All statistical analyses were performed by R (version 4.0.3; R Foundation for Statistical Computing) or MedCalc (version 15.2.2; MedCalc Software). Two-tailed *p* < 0.05 was considered statistically significant. The continuous variables were expressed as mean ± SD. Student’s *t*-test was used to compare normally distributed continuous data, and the Mann–Whitney *U*-test was used to compare non-normally distributed continuous data. The categorical variables were expressed as frequencies with percentages, and the differences in percentages were compared using the *χ*^2^ test or Fisher exact test as appropriate. The discrimination and calibration of the PTP models were assessed using the area under the receiver operating characteristic curve (AUC) and Hosmer–Lemeshow goodness-of-fit statistic (H–L *χ*^2^) according to the Transparent Reporting of a Multivariable Prediction Model for Individual Prognosis or Diagnosis (TRIPOD) statement (24). Net reclassification improvement (NRI) assessed in a reclassification table was used to determine how a PTP model-based risk assessment strategy reclassified patients into various risk groups compared with others (25). According to the reclassification table, the number needed to test was defined as the number of patients needed to be tested with the CACS-CL model to reclassify one patient from one risk group to another (26). Due to the absence of controls in the present study, the NNT was calculated as the inverse function of the difference between the correct and false reclassification rates. The Kaplan–Meier curves were generated for cumulative event-free estimates of survival from MACE and were compared by a log-rank test. The Cox proportional hazards regression models were used to calculate hazard ratios (HRs) and 95% confidence intervals (CIs), which assessed the association of risk groups to the time to the first concerned study endpoint (or censoring). For a more intuitive understanding of the clinical significance, HRs with 95% CIs were illustrated in forest plots.

## Results

### Baseline characteristics of patients

The baseline characteristics of the study population grouped by the presence of obstructive CAD are listed in Table 1. The study cohort consisted of 1,245 DM patients, of whom 44% (548/1,245) were found to have obstructive CAD on CCTA. According to the RF-CL and CACS-CL models, the average PTP was 23% and 17%, respectively. All baseline characteristics, including CACS, were significantly associated with the presence of obstructive CAD. As shown in Figure 1, of the 1,245 patients, 47% (582/1,245) and 56% (694/1,245) were assigned to the low-risk group according to the RF-CL and CACS-CL models, respectively. Table 2 shows the distribution of clinical characteristics by risk groups based on different models. There were statistically significant differences in most baseline characteristics between the two risk groups determined by the RF-CL and CACS-CL models, respectively.

**Table 1:** Baseline characteristics by presence of obstructive CAD on CCTA.

Characteristic	Total	Obstructive CAD		*p*
	*N* = 1,245	Yes (*N* = 548)	No (*N* = 697)	
Age (years, mean ± SD)	63.19 ± 10.82	66.24 ± 12.48	60.79 ± 11.05	<0.0001
Male	697 (56)	367 (67)	330 (47)	<0.0001
Hypertension	797 (64)	389 (71)	408 (58)	<0.0001
Hyperlipidemia	685 (55)	329 (60)	356 (51)	0.0020
Smoking	535 (43)	284 (52)	251 (36)	<0.0001
Family history of CAD	486 (39)	236 (43)	250 (36)	0.0115
Symptom				0.0002
Non-anginal chest pain	573 (46)	225 (41)	348 (50)	
Atypical anginal	535 (43)	246 (45)	289 (41)	
Typical anginal	137 (11)	77 (14)	60 (10)	
CACS (median, 25th–75th)	11 (0–104)	42 (0–254)	0 (0–63)	<0.0001

SD, standard deviation; CAD, coronary artery disease; CCTA, coronary computed tomographic angiography; CACS, coronary artery calcium score.

Values are presented as *n* (%) unless stated otherwise.

**Table 2:** Characteristics by risk groups based on different models.

	RF-CL model		*p*	CACS-CL model		*p*
	Low	High		Low	High	
	*n* = 582	*n* = 663		*n* = 694	*n* = 551	
Age^a^	56.9 ± 11.7	58.9 ± 10.9	<0.0001	56.8 ± 10.7	60.7 ± 11.3	<0.0001
Male	284 (49)	413 (62)	<0.0001	295 (51)	402 (61)	0.0005
Hypertension	329 (56)	468 (71)	<0.0001	341 (59)	456 (69)	0.0002
Hyperlipidemia	293 (50)	392 (59)	0.0023	298 (51)	387 (58)	0.0132
Smoking	204 (35)	331 (50)	<0.0001	217 (37)	318 (48)	0.0002
Family history of CAD	185 (32)	301 (45)	<0.0001	198 (34)	288 (43)	0.0008
Symptom			<0.0001			<0.0001
Non-anginal chest pain	314 (54)	259 (39)		305 (52)	268 (40)	
Atypical anginal	236 (41)	299 (45)		234 (40)	301 (45)	
Typical anginal	32 (5.5)	105 (16)		43 (7.4)	94 (14)	
CACS^b^	3 (0–52)	15 (0–178)	<0.0001	2 (0–81)	38 (3–292)	<0.0001

RF-CL, risk factor-weighted clinical likelihood; CACS, coronary artery calcium score; CACS-CL, CACS-weighted clinical likelihood.

Values are presented as *n* (%) unless stated otherwise.

^a^ Years, mean ± standard deviation.

^b^ Median (25th–75th).

### Comparison of the RF-Cl and CACS-CL models

**Figure 2:**
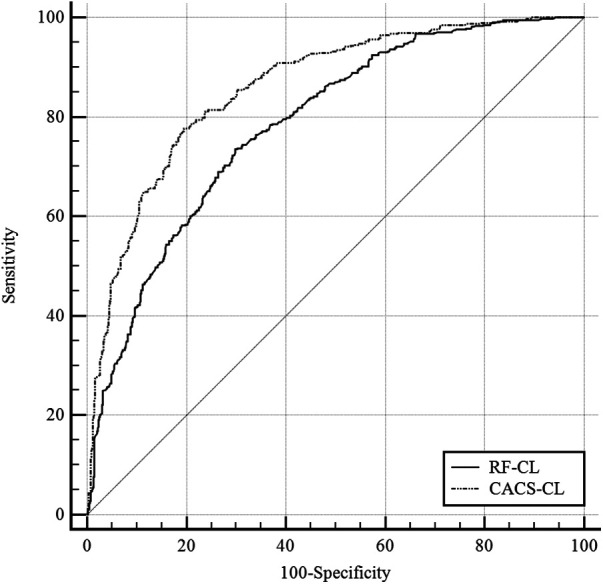
Comparison of the RF-CL and CACS-CL models by the receiver operating characteristic curves. RF-CL, risk factor-weighted clinical likelihood; CACS-CL, coronary artery calcium score-weighted clinical likelihood.

**Figure 3:**
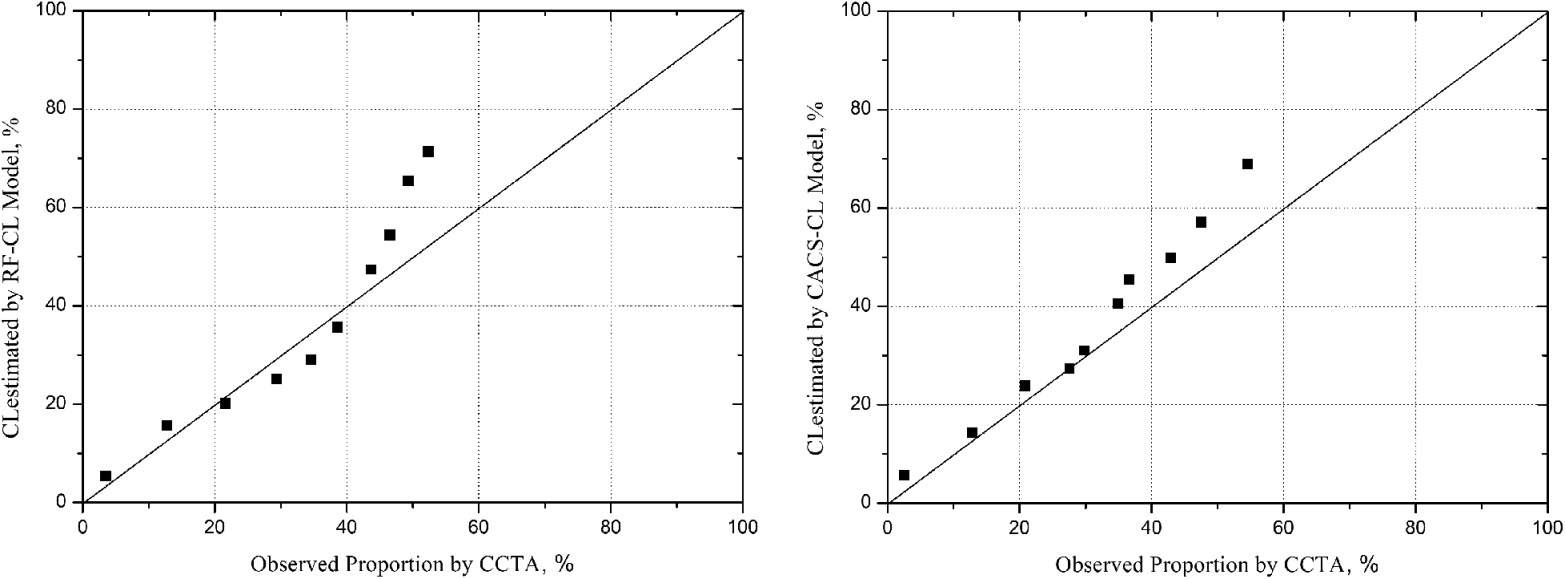
Model-specific PTP and observed probability by deciles of PTP. PTP, pretest probability; CCTA, coronary computed tomographic angiography; RF-CL, risk factor-weighted clinical likelihood; CACS-CL, coronary artery calcium score-weighted clinical likelihood.

A comparison of discrimination for the RF-CL and CACS-CL models using AUC and IDI is presented in Table 3. The AUC for the CACS-CL model was significantly larger than that for the RF-CL model (0.856 vs. 0.782, *p* = 0.0016, Figure 2). Compared with the RF-CL model, the CACS-CL model demonstrated a positive IDI (12%, *p* < 0.0001). Figure 3 illustrates the comparison of predicted and observed probabilities of obstructive CAD by deciles of PTP. Graphically, the RF-CL model (left) underestimated the probability of obstructive CAD in patients with medium PTP, with predicted values lower than those observed but overestimated the probability of obstructive CAD in patients with high PTP, with predicted values higher than those observed. As a result, the calibration of the RF-CL model was poor (H–L *χ*^2 ^= 138.52, *p* < 0.0001), but the CACS-CL model demonstrated less discordance (H–L *χ*^2 ^= 36.90, *p* < 0.0001).

**Table 3:** Discriminations of the RF-CL and CACS-CL models.

	AUC			IDI			
	Statistic	95% CI	*p* value	PTP		Statistic^b^	*p* value
				Positive^a^	Negative		
RF-CL model	0.782	0.750–0.819	0.0016	47%	21%	12%	<0.0001
CACS-CL model	0.856	0.823–0.892		54%	16%		

AUC, area under the receiver operating characteristic curve; IDI, integrated discrimination improvement; RF-CL, risk factor-weighted clinical likelihood; CACS-CL, coronary artery calcium score-weighted clinical likelihood; CI, confidence interval; CAD, coronary artery disease.

^a^ A positive patient was defined as a patient who had obstructive CAD.

^b^ Compared with the RF-CL model, the IDI of the CACS-CL model = [P(CACS-CL | Positive) − P(RF-CL | Positive)] − [ P(CACS-CL | Negative) − P(RF-CL | Negative)].

### Follow-up for study endpoints

**Figure 4:**
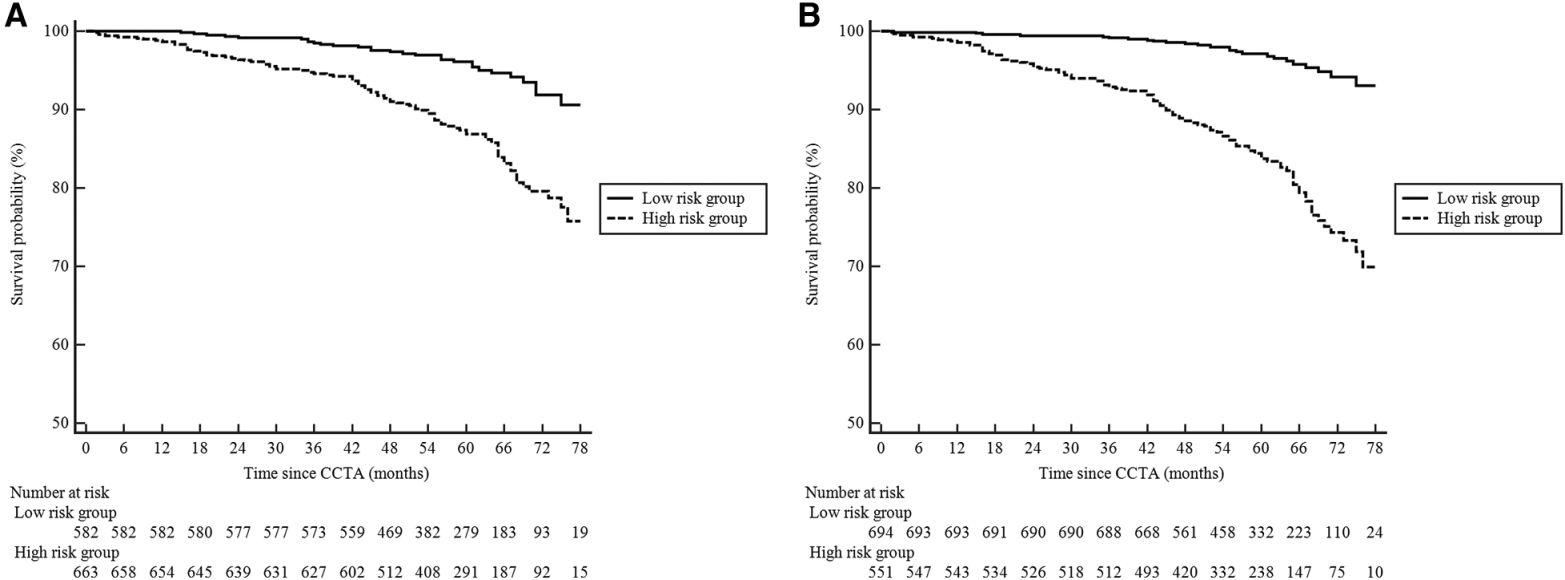
Kaplan–Meier curves comparing the high- and low-risk groups determined by the two models. (**A**) RF-CL model; (**B**) CACS-CL model. CCTA, coronary computed tomographic angiography; RF-CL, risk factor-weighted clinical likelihood; CACS-CL, coronary artery calcium score-weighted clinical likelihood.

**Figure 5:**
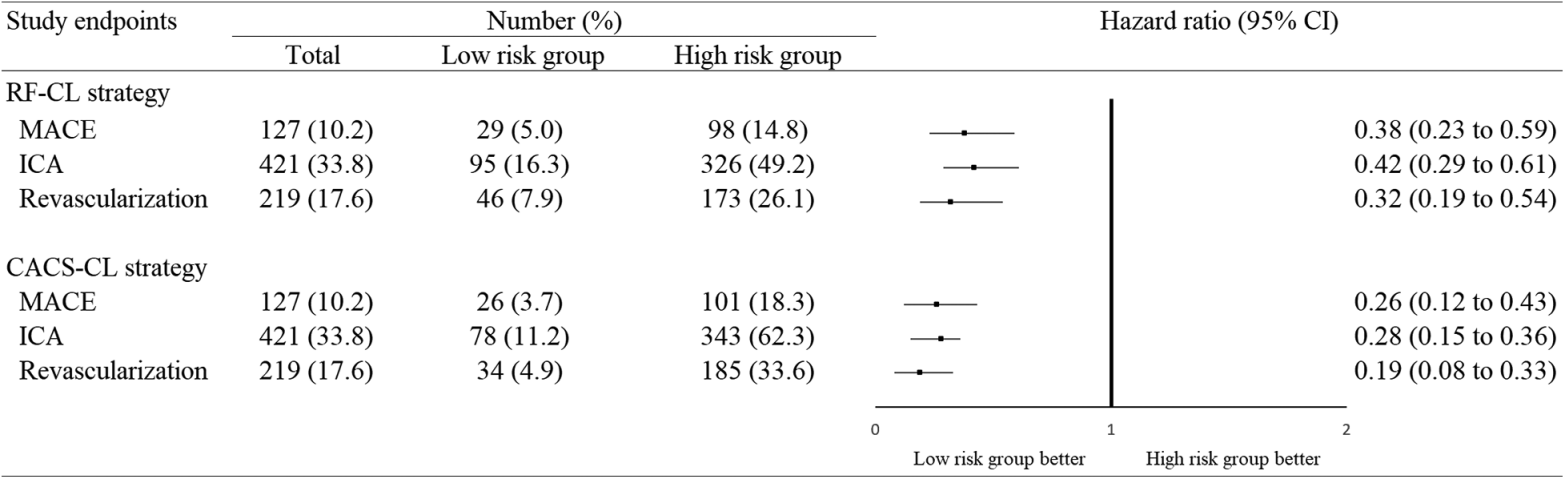
Association between study endpoints and risk groups determined by the two models. MACE, major adverse cardiovascular event; ICA, invasive coronary angiography; CI, confidence interval; RF-CL, risk factor-weighted clinical likelihood; CACS-CL, coronary artery calcium score-weighted clinical likelihood.

The patients were followed up for a median of 59 (interquartile range, 50–68) months. During the follow-up, 98 (8%) patients were lost, and 127 (10%) patients experienced MACE: 34 patients died from cardiac cause, and 93 patients suffered from non-fatal MI. Figure 4 illustrates the Kaplan–Meier estimates of patients surviving free from MACE. The high-risk group according to the RF-CL and CACS-CL models had a significantly higher risk of MACE, respectively (*p* for log-rank test, <0.0001 for the RF-CL model and <0.0001 for the CACS-CL model), but as shown in Figure 5, the association of the CACS-CL model-determined risk groups (low vs. high) with MACE was stronger than that of the RF-CL model (HR for the RF-CL model, 0.38, 95% CI: 0.23–0.59; HR for the CACS-CL model, 0.26, 95% CI: 0.12–0.43). In addition, compared with the high-risk group, the low-risk group was associated with a statistically significant reduction in utilization of invasive procedures during follow-up, according to the RF-CL model (HR for ICA, 0.42, 95% CI: 0.29–0.61; HR for revascularization, 0.32, 95% CI: 0.19–0.54) and CACS-CL model (HR for ICA, 0.28, 95% CI: 0.15–0.36; HR for revascularization, 0.19, 95% CI: 0.08–0.33), respectively (Figure 5).

### Reclassification table and NRI

Compared with patients in the high-risk group, patients in the low-risk group were less likely to have obstructive CAD [RF-CL model, 20% (118/582) vs. 66% (438/663), *p* < 0.0001; CACS-CL model, 9% (62/694) vs. 88% (486/551), *p* < 0.0001]. Table 4 is the reclassification table comparing the CACS-CL with the RF-CL model. Compared with the RF-CL model, the CACS-CL model correctly divided 177 patients from the high- to low-risk group but falsely divided 9 patients from the low- to high-risk group in 697 negative patients. Of the 548 positive patients, 61 were correctly divided into the high-risk group but falsely divided 5 into the low-risk group. In other words, to avoid an unnecessary CIT and omissive CIT, the number needed to test CACS was 1,245/(177-9) ≈ 8 and 1,245/(61-5) = 21, respectively. Thus, compared with the RF-CL model, the NRI for the CACS-CL model was 24% in negative, 10% in positive, and 34% in all (*p* < 0.0001). The application of the CACS-CL model instead of the RF-CL model would result in a prominent change in downstream clinical management: 24% of the negative patients were reclassified into the low-risk group, for which no further CIT was recommended; 10% of the positive patients were reclassified into the high-risk group, for which further CIT recommended.

**Table 4:** Reclassification table comparing the RF-CL and CACS-CL models.

	Risk groups by CACS-CL model			Reclassification^a^		NRI^b^	*p*
	Low	High	Total	Up	Down		
Risk groups by RF-CL model							
Negative patients				1.29%	25.39%	34.32%	<0.0001
Low	455	9	464				
High	177	56	233				
Total	632	65	697				
Positive patients^c^				11.13%	0.91%		
Low	57	61	118				
High	5	425	438				
Total	62	486	548				

RF-CL, risk factor-weighted clinical likelihood; CACS-CL, coronary artery calcium score-weighted clinical likelihood; CAD, coronary artery disease.

^a^ The classification of patients by the CACS-CL model was compared with that by the RF-CL model.

^b^ NRI = [P(Up | Positive) − P(Down | Positive)] − [ P(Up | Negative) − P(Down | Negative)].

^c^ A positive patient was defined as a patient who had obstructive CAD.

## Discussion

In this CCTA-based cohort comprised of patients with DM and SCP suspected of CCS, the CACS-CL model revealed a larger AUC, less discrepancy between observed and predicted probabilities, and a positive IDI and NRI when compared with the RF-CL model. Both the RF-CL and CACS-CL models classified a proportion of patients into the low-risk groups with low prevalence of CAD and MACE, but the incorporation of CACS in the CACS-CL model was associated with improved effectiveness of risk assessment to optimize downstream clinical management.

Although all complications of DM are important, CAD specifically continues to be the leading cause of morbidity and mortality in this group (2, 27). In addition to being associated with increased cardiovascular risk, DM has the potential to affect a number of clinical management choices for CAD, including whether or not a CIT is appropriate (2, 27). However, two large randomized controlled trials (RCTs), FACTOR-64 and DIAD, did not support routine screening for CAD by CIT in DM patients to reduce MACE (6, 7). In this context, a PTP estimation-based approach for risk assessment to identify individuals who may derive maximum benefit from further CIT in patients with DM and SCP has been recommended by the newest guidelines (2).

Both the RF-CL and CACS-CL models were externally validated and compared in different CCTA-based cohorts of general SCP patients and our previous studies from the CICM-SCP registry (10–13). Furthermore, we have demonstrated that the RF-CL model was associated with greater efficiency in optimizing downstream clinical management for patients with SCP and DM (9), which was also supported by the lower prevalence of CAD and MACE when comparing the low- with the high-risk group determined by the RF-CL model in this study. However, the modest AUC and evident discrepancy between observed and predicted probabilities for the RF-CL model implied that there was still room for improvement in terms of the PTP estimation for patients with SCP and DM. Moreover, a cumulative MACE rate of approximately 5% is not prerequisite enough for allowing a more restrictive policy to investigate CAD in the low-risk group, especially considering the subsequent intervention after CCTA in a real-world cohort.

CACS revealed robust diagnosis and prognosis information above other biomarkers (28–31) and was recommended by recent guidelines especially for patients at low risk (8, 14), but our recent works have demonstrated that the performance of CACS alone was not as good as that of CACS-CL model (11, 32). In conformity with previous findings comparing the CACS-CL model with the RF-CL model (10–13), we demonstrated that the CACS-CL model offered a more accurate estimation of PTP and prediction of MACE. As far as we know, this is the first comparative description of the RF-CL and CACS-CL models in patients with SCP and DM. Moreover, there was a significant increase in clinically useful reclassification when CACS was added to these patients: when comparing the CACS-CL with the RF-CL model, the NRI was positive, and the association between study endpoints (MACE, use of downstream diagnostic and therapeutic interventions) and risk groups was enhanced. The replacement of the RF-CL model with the CACS-CL model would avoid an unnecessary CIT and omissive CIT at the expense of 8 and 21 additional CACS scans, respectively. Thus, considering the CACS scan as a quick, lower-radiation, and relatively inexpensive tool, more emphasis should be placed on the CACS-CL model for the effective identification of patients with SCP and DM who may derive minimal benefit from further CIT in clinical practice, which has been supported by studies evaluating the role of multimodal imaging in CCS (33–35).

Although this is the first study to evaluate the RF-CL and CACS-CL models for patients with SCP and DM, several issues merit consideration. First, this study was an observational cohort. The clinical management of patients before and after CCTA relied on a local physician. Thus, whether the CACS-CL model will lead to more appropriate decision-making of downstream management and better clinical outcomes needs to be addressed in future studies, such as RCTs. Second, this analysis focused on the presence of obstructive CAD documented by CCTA. Previous studies have demonstrated that CCTA had a high negative predictive value compared with ICA (36, 37), so CCTA could offer robust reassurance to exclude obstructive CAD. Moreover, we defined unassessable segments as positive ones based on current guideline recommendations in which further testing should be referred for non-conclusive CCTA. Third, this analysis focused on the presence of coronary diameter stenosis ≥50%. Evaluation of high-risk CAD, such as left main disease or three-vessel disease with a maximal degree of coronary diameter stenosis ≥70%, would be helpful to identify patients who may derive maximal benefit from revascularization (38–40). Fourth, the potential advantage of CACS is its ability to reclassify patients with borderline PTP (5%–15%), which has been demonstrated in the general population (12, 26, 41). Thus, further studies are needed to investigate whether the reclassification effect of CACS is most pronounced for diabetic patients with borderline PTP. Lastly, this study only focused on SCP. Thus, the conclusions should not be extrapolated to patients with dyspnea or acute chest pain or asymptomatic patients.

## Conclusions

Among patients with DM and SCP suspected of CCS, the CACS-CL model demonstrated superiority in terms of the diagnosis of obstructive CAD and prediction of MACE. As a result, compared with the RF-CL model, the CACS-CL model-based risk assessment might have more potential to avoid unnecessary and omissive CIT at a low expense. In the future, the cost-effectiveness of the CACS-CL model that increases the use of CACS scans needs to be comprehensively validated in RCTs comprised of patients with DM and SCP suspected of CCS.

## Data Availability

The raw data supporting the conclusions of this article will be made available by the authors, without undue reservation.
