# Supplementary material for: Comparison of the RF-CL and CACS-CL models to estimate the pretest probability of obstructive coronary artery disease and predict prognosis in patients with stable chest pain and diabetes mellitus
Source: Front Cardiovasc Med. 2024 Mar 22;11:1368743. doi: 10.3389/fcvm.2024.1368743 (PMC10995235; doi:10.3389/fcvm.2024.1368743)
Supplement: Supplementary file 1 [file Table1.docx]

**Supplemental Methods**

**Study population**

The Departments of Cardiology in Tianjin University Chest Hospital (Tianjin, China) and Heart Center in Beijing Chaoyang Hospital, Capital Medical University (Beijing, China) are both important cardiac centers recognized as tertiary A level in Beijing-Tianjin-Hebei region, which has more than 100 million inhabitants. The totally annual number of patients referred for assessment of stable chest pain (SCP) in two centers is more than 20,000. Since the last decade, coronary CT angiography (CCTA) has gradually become the preferred first-line imaging testing for patients with SCP in two centers. Thus, from January 2016, we conducted the CCTA Improves Clinical Management of Stable Chest Pain registry, a prospective, natural history and dynamic cohort which included consecutive participants who were referred to CCTA for the assessment of SCP. The ongoing registry includes cross-sectional findings as well as longitudinal follow-up of clinical outcomes and partial information about this cohort has been [describe](javascript:;)d previously[[1-3](#_ENREF_1)].

**Baseline clinical data collection**

Hypertension was defined as blood pressure of ≥140/90 mmHg or the use of anti-hypertension medication. Hyperlipidemia was defined as total cholesterol of ≥220 mg/dL, low-density lipoprotein cholesterol of ≥140 mg/dL, fasting triglycerides of ≥150 mm/dL or receiving treatment with oral lipid-lowering agents. Smoking was defined as current smoking or smoking in past 6 months. A family history of CAD was defined as diagnosis of the disease in a male first-degree relative before 55 years of age or in a female first-degree relative before 65 years of age. Typical angina was defined as having 3 characteristics: 1) substernal discomfort of characteristic quality, 2) precipitated by physical exertion or emotion, and 3) relieved with rest or nitroglycerin within 10 min. Atypical angina was defined as having 2 of the 3 definition characteristics. Nonanginal chest pain was characterized as chest pain or discomfort that meets 1 or 0 of the 3 definition characteristics[[4](#_ENREF_4)].

**CACS and CCTA**

Before every CCTA, a noncontrast cardiac CT was acquired to quantify CACS using standard techniques. With a slice thickness of 3 mm and a prospective electrocardiogram triggering technique, coronary calcium was present if at least four contiguous pixels with an attenuation of ≥130 Hounsfield Units (HU) were detected along a coronary artery and CACS of all coronary arteries was calculated as previously described by Agatston[[5](#_ENREF_5)]. Two investigators, a radiologist and a cardiologist, who were blinded to clinical data and results of CCTA performed the quantitative CACS analyses by a semi-automated software (Syngo Calcium Scoring, Siemens Medical Solutions, German).

All CCTA scans were performed with a second-generation dual-source CCTA scanner (Somatom Definition Flash, Siemens Medical Solutions, Forchheim, Germany). Sublingual nitroglycerine and heart-rate control by betablocker for a target heart rate ≤70 beats/min were administered as appropriate. Afterwards, A contrast enhanced CCTA was performed with detector collimation of 2×128×0.6 mm, slice thickness of 0.6 mm, gantry rotation time of 280 ms, heart rate adaptive pitch of 0.2–0.5, tube current of 290 to 560 mAs/rotation and tube voltage of 80–120 kV. Contrast volume was 60–90 mL followed by a normal saline of 50 mL and was injected intravenously in an antecubital vein. Bolus tracking is used to synchronize the arrival of contrast in the coronary arteries and the initiation of the scan, and the region of interest (ROI) was set at the root of the ascending aorta. Data acquisition was initiated with a delay of 5 seconds after signal attenuation threshold (100 HU) was reached in ROI. Image scan was triggered from 30 % to 80 % of the R-R interval. Three experienced observers, two radiologist and a cardiologist, who were blinded to the clinical data and CACS evaluated the CCTA data on a Syngo Multimodality workstation (Siemens, German).

Each coronary segment with a >2mm diameter was analyzed for the presence of coronary diameter stenosis. According to the Coronary Artery Disease – Reporting and Data System[[6](#_ENREF_6)], the maximal degree of coronary diameter stenosis was defined as no CAD (0%), nonobstructive CAD (1-49%) and obstructive CAD (≥50%). Interobserver disagreements were resolved by consensus.

**Follow-up**

Contact information of all patients including telephone number, e-mail address and home address were collected before CCTA. All patients were followed up until March 2023 and follow-up information was obtained by phone call or physician interview as appropriate. All endpoints were adjudicated via retrieve of follow-up information and medical records by an independent clinical event committee who was blinded to other data.

**References**

1. Zhou J, Li C, Cong H, Duan L, Wang H, Wang C, Tan Y, Liu Y, Zhang Y, Zhou X, Zhang H, Wang X, Ma Y, Yang J, Chen Y, Guo Z. Comparison of Different Investigation Strategies to Defer Cardiac Testing in Patients With Stable Chest Pain. J Am Coll Cardiol Img. 2022;15:91-104.

2. Zhou J, Chen Y, Zhang Y, Wang H, Tan Y, Liu Y, Huang L, Zhang H, Ma Y, Cong H. Epicardial Fat Volume Improves the Prediction of Obstructive Coronary Artery Disease Above Traditional Risk Factors and Coronary Calcium Score. Circ Cardiovasc Imaging. 2019;12:e008002.

3. Zhou J, Li C, Zhang H, Liu C, Yang J, Zhao J, Hou Y, Tan Y, Wang H, Li Y, Xie C, Wang M, Wang C, Zhang E, Wang S, Zhao P, Shan D, Liang S, Gao Y, Huo Y, Cong H, Guo Z, Chen Y. Association between Coronary Artery Disease Reporting and Data System–recommended Post–Coronary CT Angiography Management and Clinical Outcomes in Patients with Stable Chest Pain from a Chinese Registry. Radiology. 2023;307:e222965.

4. Diamond GA. A clinically relevant classification of chest discomfort. J Am Coll Cardiol. 1983;1:574-5.

5. Agatston AS, Janowitz WR, Hildner FJ, Zusmer NR, Viamonte M, Detrano R. Quantification of coronary artery calcium using ultrafast computed tomography. Journal of the American College of Cardiology. 1990;15:827-32.

6. Cury RC, Abbara S, Achenbach S, Agatston A, Berman DS, Budoff MJ, Dill KE, Jacobs JE, Maroules CD, Rubin GD, Rybicki FJ, Schoepf UJ, Shaw LJ, Stillman AE, White CS, Woodard PK, Leipsic JA. CAD-RADS(TM) Coronary Artery Disease - Reporting and Data System. An expert consensus document of the Society of Cardiovascular Computed Tomography (SCCT), the American College of Radiology (ACR) and the North American Society for Cardiovascular Imaging (NASCI). Endorsed by the American College of Cardiology. J Cardiovasc Comput Tomogr. 2016;10:269-81.
